# On the hydrolysis of diethyl 2-(perfluorophenyl)malonate

**DOI:** 10.3762/bjoc.16.153

**Published:** 2020-07-28

**Authors:** Ilya V Taydakov, Mikhail A Kiskin

**Affiliations:** 1 P.N. Lebedev Physical Institute of the Russian Academy of Sciences. Leninskiy prospect, 53, Moscow, GSP-1, 119991, Russian Federation; 2 G.V. Plekhanov Russian University of Economics, Stremyanny per. 36, Moscow, 117997, Russian Federation; 3 N.S. Kurnakov Institute of General and Inorganic Chemistry, Russian Academy of Sciences, Leninskiy prospect, 31, Moscow, GSP-1, 119991, Russian Federation

**Keywords:** decarboxylation, fluorinated aromatic compounds, hydrolysis of esters, 2-(perfluorophenyl)acetic acid, 2-(perfluorophenyl)malonic acid

## Abstract

Diethyl 2-(perfluorophenyl)malonate was synthesized in 47% isolated yield by the reaction of sodium diethyl malonate and hexafluorobenzene. The resulting compound was considered as a starting material for synthesizing 2-(perfluorophenyl)malonic acid by hydrolysis. It was found that the desired 2-(perfluorophenyl)malonic acid could not be obtained from this ester by hydrolysis, neither under basic nor under acidic conditions. Nevertheless, hydrolysis of the ester with a mixture of HBr and AcOH gave 2-(perfluorophenyl)acetic acid in a good preparative yield of 63%. A significant advantage of this new approach to 2-(perfluorophenyl)acetic acid is that handling toxic substances such as cyanides and perfluorinated benzyl halides is avoided.

## Introduction

**Figure 1:**
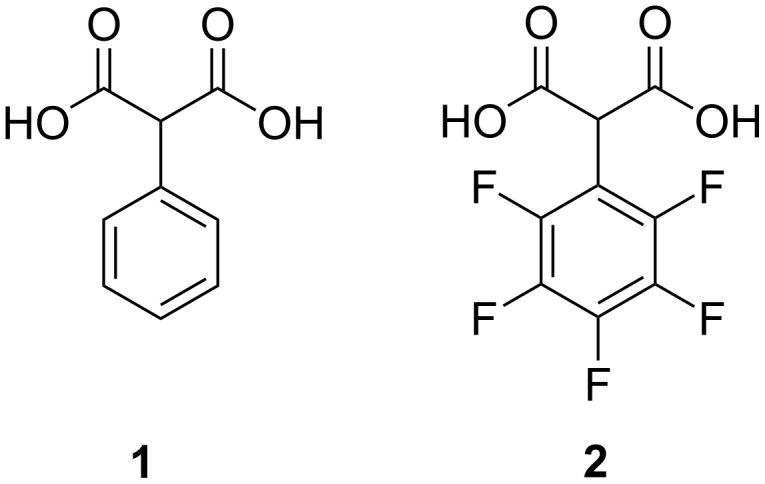
Phenylmalonic acids.

2-Phenylmalonic acid (**1**) and its esters are useful and versatile intermediates in the synthesis of many practically important compounds, e.g., pharmacologically active substances [1–5], in asymmetric synthesis and catalysis [6–8], as precursors of heterocyclic compounds [9–11], as ligands in coordination chemistry [12–16] and for other applications [17–18]. Incorporation of fluorine atoms into an organic molecule is known to be one of the most powerful tools for fine tuning of chemical and physical properties [19–20]. For example, thermal stability, solubility, metabolic and oxidative stability, lipophilicity, membrane permeability, complexing ability, acid-base, and many other properties can be modified in a broad range by fluorination (directly or indirectly) of initial organic molecules. In the ongoing project, we are interested in synthesizing 2-(perfluorophenyl)malonic acid (**2**, Figure 1) as a new ligand for the preparation of 3d and 4f heterometallic coordination compounds.

## Results and Discussion

**Scheme 1:**
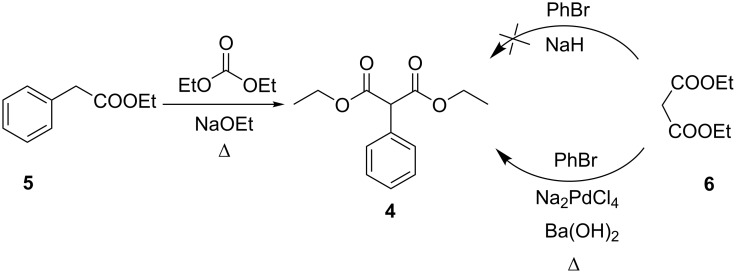
Synthesis of diethyl 2-phenylmalonate (**4**).

To our surprise, 2-(perfluorophenyl)malonic acid (**2**) was not described in the literature to date. The only reference to this compound actually concerned the formation of the anion from diethyl 2-(perfluorophenyl)malonate (**3**) with excess NaH [21]. At the same time, diethyl 2-(perfluorophenyl)malonate (**3**) is a readily accessible compound. Diethyl 2-phenylmalonate (**4**) is usually obtained (Scheme 1) by condensation of ethyl phenylacetate (**5**) and diethyl carbonate under basic conditions [22] because of the low reactivity of bromobenzene in noncatalytic nucleophilic reactions with sodium salts of diethyl malonate (**6**) [23–24].

**Scheme 2:**
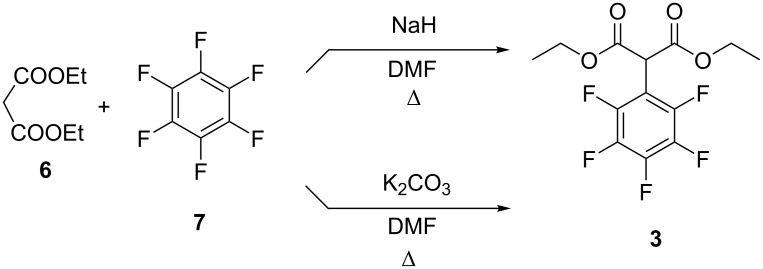
Synthesis of diethyl 2-(perfluorophenyl)malonate (**3**).

In contrast with bromobenzene, hexafluorobenzene (**7**) is sufficiently reactive in nucleophilic substitution reactions, and thus the synthesis of 2-(perfluorophenyl)malonate (**3**) is straightforward. A synthesis of diethyl 2-(perfluorophenyl)malonate (**3**) was first described in patent literature [25]. The target compound was obtained by the reaction of diethyl malonate, NaH and C_6_F_6_ in DMF for 5 h under reflux. This method was modified by Vlasov et al. [26], then NaH was replaced [27] by anhydrous K_2_CO_3_ and the reaction temperature was decreased [28] to 60 °C (Scheme 2). Although the latter method claimed to give diethyl 2-(perfluorophenyl)malonate (**3**) in 92% yield, it can hardly be scaled up due to the utilization of gradient column chromatography for the separation of the desired product. We obtained diethyl 2-(perfluorophenyl)malonate (**3**) in 47% isolated yield by a modified method [26]. The product was isolated on 150 mmol scale by simple vacuum distillation.

Hydrolysis of diethyl 2-(perfluorophenyl)malonate (**3**) unexpectedly turned out to be quite challenging. Unsubstituted diethyl or dimethyl 2-phenylmalonate can be readily hydrolyzed under basic conditions by heating with aqueous or mixed water–EtOH solutions [29] or in biphasic water–Et_2_O mixtures [30] under reflux conditions.

**Figure 2:**
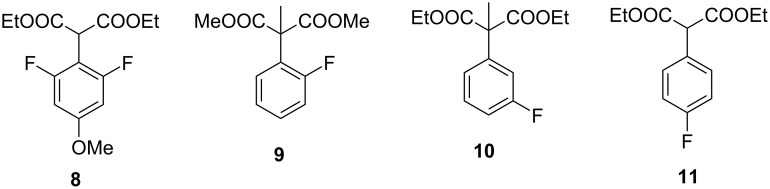
Esters of fluorine-substituted 2-phenylmalonic acids.

An exhaustive analysis of the literature revealed that virtually the same conditions were suitable for the hydrolysis of diethyl 2‐(2,6‐difluoro‐4‐methoxyphenyl)malonate (**8**) [31], dimethyl 2-(2-fluorophenyl)-2-methylmalonate (**9**) [32], diethyl 2-(3-fluorophenyl)-2-methylmalonate (**10**) [33], and diethyl 2-(4-fluorophenyl)malonate (**11**) [6,32] (Figure 2) with minor variations in the alkali concentration and temperature. No nucleophilic substitution of activated fluorine atoms or other side reactions were observed under the conditions mentioned above.

**Scheme 3:**
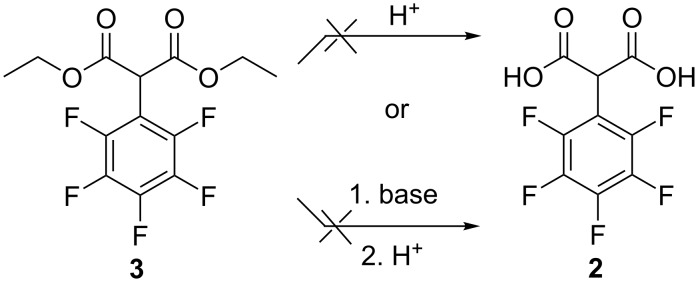
Hydrolysis of diethyl 2-(perfluorophenyl)malonate (**3**).

In preliminary experiments, we tested the mildest system – biphasic 10% KOH aqueous solution–Et_2_O at reflux temperature (35 °C) and vigorous agitation for the hydrolysis of diethyl 2-(perfluorophenyl)malonate (Scheme 3)

No reaction occurred under these conditions after 5 h of refluxing. Increasing the alkali concentration up to 20% did not induce a reaction. Different reaction conditions and bases were used, and the results are summarized in Table 1.

**Table 1:** Hydrolysis of **3** under basic conditions.

Base	Solvent(s)	Time, h	Temperature, °C	Yield of compound^a^	

				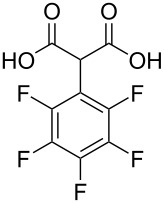 **2**	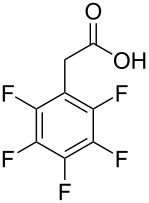 **12**

KOH (5 equiv)	10% H_2_O + Et_2_O	5	reflux (35)	n.d.^b^	n.d.
KOH (5 equiv)	20% H_2_O + Et_2_O	5	reflux (35)	n.d.	n.d.
NaOH (3 equiv)	15% H_2_O	15	20	n.d.^b^	n.d.
NaOH (3 equiv)	15% H_2_O	5	80	n.d.	12^c^
NaOH (3 equiv)	15% H_2_O + EtOH (1:2 v/v)	15	20	n.d.^b^	n.d.
NaOH (3 equiv)	15% H_2_O + EtOH (1:2 v/v)	5	reflux (80)	n.d.^b^	15^c^
LiOH (2 equiv)	dioxane	15	20	n.d.^b^	n.d.
LiOH (2 equiv)	dioxane–H_2_O (10%)	15	20	n.d.^b^	11^c^
LiOH (2 equiv)	dioxane–H_2_O (10%)	5	80	n.d.	17^c^

^a^n.d. – not detected by NMR, see Supporting Information File 1; ^b^the starting material (ester **3**) was recovered; ^c^isolated yield;

In general, one can conclude that under mild reaction conditions (room temperature, biphase mixtures) the starting ester remained intact, while under drastic conditions (high concentration of alkali, homogeneous solutions, elevated temperatures), decomposition and/or decarboxylation occurred. However, it is possible that decarboxylation took place during the isolation of the free acid. In all cases, no desired malonic acid **2** was isolated from the reaction mixtures; the main part of the original material was recovered. In some experiments, variable amounts of 2-(perfluorophenyl)acetic acid (**12**) were obtained after acidification of the basic solution. Moreover, noticeable decomposition of **3** was observed along with the formation of acid **12**. The nature of these byproducts was not analyzed. Probably, they are formed by nucleophilic substitution of fluorine atoms in the perfluorophenyl moiety.

**Figure 3:**
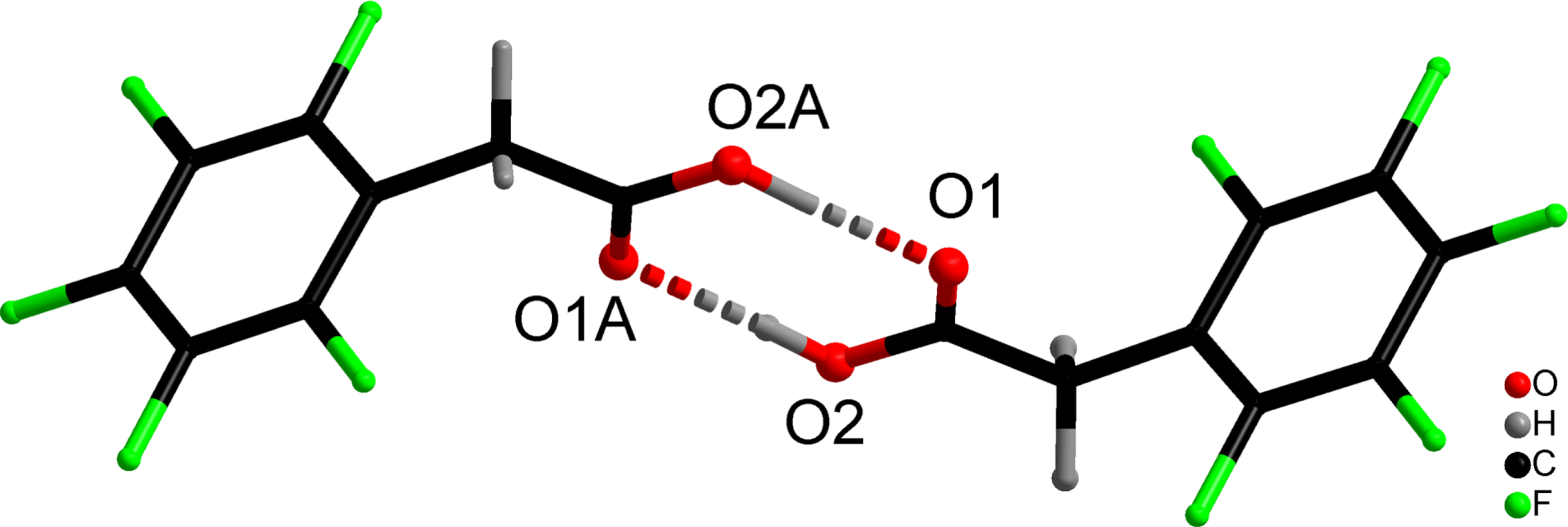
Molecular structure of 2-(perfluorophenyl)acetic acid (**12**).

Acid **12** is fairly soluble in water and the separation of reaction products is cumbersome (see Supporting Information File 1). The structure of acid **12** in solid form was studied by single crystal X-ray diffraction experiments (Figure 3). The structural parameters were deposited at CCDC (deposit No. 1993963, see Supporting Information File 1 for details).

**Scheme 4:**
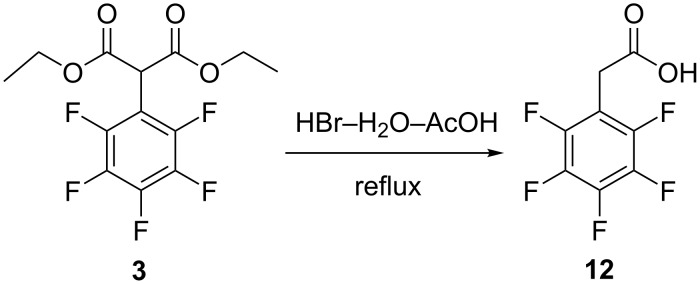
Formation of 2-(perfluorophenyl)acetic acid (**12**).

Since basic conditions seem to be unsuitable for the hydrolysis of diethyl 2-(perfluorophenyl)malonate, acid-catalyzed reactions were also tested. Trifluoroacetic acid (TFA) is a common reagent for the catalytic cleavage of *tert*-butyl esters, but in the stoichiometric quantity it is also suitable for the cleavage of nonvolatile methyl or ethyl esters due to transesterification [34–35]. The removal of highly volatile methyl or ethyl trifluoroacetates from the reaction mixture is the driving force of this process. Unfortunately, 2-(perfluorophenyl)malonate does not react with excess TFA (up to 10 equivalents) even under reflux conditions, but in one experiment a catalytic amount of concentrated H_2_SO_4_ was added, and then coincidentally the reaction mixture was slightly overheated (to 100 °C), which led to the evaporation of the entire acid. The remaining solid was identified as 2-(perfluorophenyl)acetic acid (**12**). We believe that 2-(perfluorophenyl)acetic acid might be formed by thermal decarboxylation of the desired 2-(perfluorophenyl)malonic acid. To prove this hypothesis, 2-(perfluorophenyl)malonate (**3**) was heated under reflux conditions with an excess of 48% aqueous HBr solution according to the method described in [36], but only traces of 2-(perfluorophenyl)acetic acid (**12**) were isolated after 16 h of reflux along with the unchanged original ester. In addition, considerable darkening of the biphase reaction mixture was observed. To overcome the problem of miscibility of the ester and acid, AcOH was used as a co-solvent. After some optimization of the reaction conditions, it was found that best results were achieved if a 1:5 v/v mixture of 48% HBr and glacial AcOH was used. The starting ester is fully soluble in this mixture, and the reaction occurs in a homogeneous solution (Scheme 4).

Unfortunately, in all experiments hydrolysis was accompanied by complete decarboxylation, and only 2-(perfluorophenyl)acetic acid (**12**) was separated from the reaction mixtures. The results of the acidic hydrolysis are summarized in Table 2. It seems that 2-(perfluorophenyl)malonic acid (**2**) is unexpectedly thermally unstable, because as a rule, decarboxylation of other phenylmalonic acids with strong electron-withdrawing groups required much higher temperatures (160–200 °C) or the presence of a catalyst [31,37].

**Table 2:** Hydrolysis of **3** under acidic conditions.

Acid	Solvent(s)	Time, h	Temperature, °C	Yield of compound^a^	

				**2**	**12**

TFA (5 equiv)	CH_2_Cl_2_	15	20	n.d.^b^	n.d.
TFA (10 equiv)	neat	15	20	n.d.^b^	n.d.
TFA (5 equiv)	neat	6	reflux (75)	n.d.^b^	trace
TFA (5 equiv)	neat + 1 drop of conc. H_2_SO_4_	10	reflux (75)	n.d.^b^	trace
TFA (5 equiv)	neat + 1 drop of conc. H_2_SO_4_	5	reflux (75) + overheat (100)	n.d.	35^c^
HBr (25 equiv)	48% H_2_O	15	100	n.d.^b^	10^c^
HBr (25 equiv)	48% H_2_O + AcOH (1:2 v/v)	15	20	n.d.^b^	n.d.
HBr (6 equiv)	48% H_2_O + AcOH (1:5 v/v)	10	reflux (120)	n.d.	63^c^

^a^n.d. – not detected by NMR, see Supporting Information File 1; ^b^the starting material (ester **3**) was recovered; ^c^isolated yield;

However, acid-catalyzed hydrolysis of diethyl 2-(perfluorophenyl)malonate (**3**) can be used as a convenient, inexpensive and simple multigram approach to 2-(perfluorophenyl)acetic acid (**12**). This new method is shorter and much safer than the classical one based on the hydrolysis of 2-(perfluorophenyl)acetonitrile [38–39], because utilization of both toxic 1-(bromomethyl)-2,3,4,5,6-pentafluorobenzene and alkali metal cyanides is avoided.

## Conclusion

To summarize, we have extensively investigated the reactivity of diethyl 2-(perfluorophenyl)malonate (**3**) towards hydrolysis in acidic and basic media. It was revealed, that the ester is fairly stable in basic and acidic solutions at ambient temperatures and decompose to a mixture of products (2-(perfluorophenyl)acetic acid (**12**) was identified as a major product) at harsh basic conditions. Vigorous hydrolysis by a mixture of aqueous HBr and AcOH at reflux temperature led to the formation of 2-(perfluorophenyl)acetic acid (**12**) as single product in good preparative yield. Evidently, ethyl 2-(perfluorophenyl)malonate (**3**) is not suitable for the preparation of 2-(perfluorophenyl)malonic acid (**2**), due to its thermal instability and strong tendency to decarboxylation. We believed, that di-*tert*-butyl 2-(perfluorophenyl)malonate or dibenzyl 2-(perfluorophenyl)malonate, which are cleaving under very mild conditions are better precursors, but these esters are expensive, hardly accessible and can barely be used for large-scale preparation of 2-(perfluorophenyl)malonic acid (**12**).

## Supporting Information

File 1 Detailed information about experimental procedures, X-ray diffraction experiments for compound **12** and characterization data for compounds **3** and **12**.
