# Supplementary material for: On the hydrolysis of diethyl 2-(perfluorophenyl)malonate
Source: Beilstein J Org Chem. 2020 Jul 28;16:1863–8. doi: 10.3762/bjoc.16.153 (PMC7404147; doi:10.3762/bjoc.16.153)

BEILSTEIN JOURNAL OF ORGANIC CHEMISTRY

Supporting Information

for

On the hydrolysis of diethyl 2-(perfluorophenyl)malonate

Ilya V. Taydakov and Mikhail A. Kiskin

Beilstein J. Org. Chem. **2020,** *16,* 1863–1868. doi:10.3762/bjoc.16.153

Detailed information about experimental procedures, X-ray diffraction experiments for compound 12 and characterization data for compounds 3 and 12

License and Terms: This is a supporting information file under the terms of the Creative Commons Attribution License (http://creativecommons.org/licenses/by/4.0). Please note that the reuse, redistribution and reproduction in particular requires that the authors and source are credited.
The license is subject to the *Beilstein Journal of Organic Chemistry* terms and conditions: (https://www.beilstein-journals.org/bjoc)

Table of Contents:

General information: S2

General procedures for preparation of **3** and **12** S3

Molecular and crystal structure of compound **12** S5

References S6

Characterization data (**3** and **12**) S7

General information

Sodium hydride (60% dispersion in mineral oil), diethyl malonate and other common chemicals were purchased from Acros Organics (Belgium). Hexafluorobenzene was purchased from SIA "P&M-Invest" Ltd. (Moscow, Russia) and distilled before use. DMF was distilled over CaH_2 under an Ar atmosphere just before use. Oven-dried glassware was used for the arylation reaction and manipulations were conducted under dry Ar blanket.

Caution ! Dry or oil-free sodium hydride is highly pyrophoric and reacts violently with water. It should never be exposed to air during the washing procedure. It must be wetted by a solvent at all times and inert atmosphere must be maintained during the manipulations. Traces of NaH in hexane after the washing procedure must be decomposed by careful addition of anhydrous ethanol followed by 96% ethanol before collecting it in a waste container.

NMR spectra were recorded at ambient temperature on a Bruker AM-300 instrument operating at 300, 282.5 and 75.5 MHz for ^{1}H, ^{19}F and ^{13}C nuclei, respectively. Chemical shifts were referenced to $CHCl_3$ (7.26 ppm), $CDCl_3$ (77.2 ppm) and $CFCl_3$ (0.00 ppm) for ^{1}H, ^{13}C and ^{19}F spectra, respectively. ^{13}C NMR spectra were also recorded on a Bruker DXR-500 instrument operated at 125.8 MHz. FTIR spectra were obtained on a Perkin-Elmer system Spectrum One 100 FTIR spectrometer in KBr pellets. HRMS was performed on a Bruker MicroTOF instrument with an ESI ionization source in the positive ion detection mode. Melting points were measured on a Büchi Melting Point M-560 apparatus and were not corrected. Elemental microanalysis was performed at the Laboratory of microanalysis of Nesmeyanov Institute of Organoelement Compounds, Moscow, Russia.

Synthetic procedures and analytical data for compounds 3 and 12.

Diethyl 2-(perfluorophenyl)malonate (3)

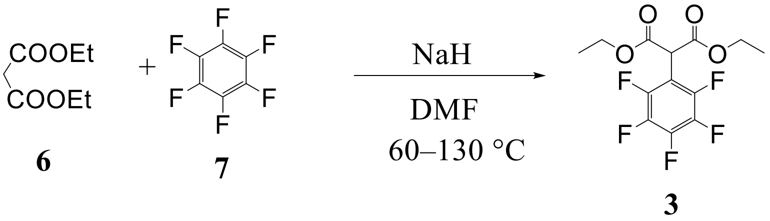

This compound was a obtained by a modified method of Vlasov [1]. Sodium hydride (60% dispersion in mineral oil), 6.8 g (170 mmol) was placed into a 500 mL round bottom flack equipped with an Ar inlet, magnetic stirring bar, dropping funnel and reflux condenser. The open end of the condenser was connected to a bubble counter filled with mineral oil. Dry hexane (50 mL) was added to NaH and the resulting suspension was stirred for a short time with a magnetic stirring bar and left until the hexane layer became clear (20–30 min). Hexane was cautiously removed with a glass pipette until a wet paste of NaH remained. This procedure was repeated twice. After that, 90 mL of dry DMF was added to NaH, and diethyl malonate (150 mmol, 24.1 g, 23 mL) was added dropwise with continuous stirring. When gas evolution ceased, hexafluorobenzene (155 mmol, 28.8 g, 18 mL) was added slowly, and the resulting suspension was heated to a gentle reflux. Heating was continued for 10 h. At the end of the reaction, the temperature of the heating bath was around 135 °C. The reaction mixture was cooled to room temperature and poured into 120 mL of 5% HCl solution. The product was extracted with 4 × 50 mL of EtOAc. The organic phase was washed with 3 × 10 mL portions of water to a neutral pH, then with 10 mL of brine, and then dried over $MgSO_4$. The solvent was then removed in a rotary evaporator (bath temperature 45 °C). The clear oily residue was transferred into a Claisen flack and vacuum distilled through a short Vigreux column. The initial pressure was 50 Torr, and the fraction with boiling temperatures up to 120 °C (mainly unreacted diethyl malonate) was collected. The pressure was decreased to 2 Torr and the fraction with a boiling point of 114–117 °C (lit. 116–118 °C at 1.5 Torr [1]) was collected. The yield of diethyl 2-(perfluorophenyl)malonate (**3**) as a clear liquid was 22.9 g (47%). The analytical sample was obtained by repeated distillation.

^{1}H NMR ($CDCl_3$, 300 MHz): δ = 4.95 (s, 1H, CH), 4.25(q, 2H, J=7.1Hz, CH_2), 1.31(t, 3H, J=7.1Hz,CH_3),

^{19}F NMR ($CDCl_3$, 282.5 MHz): δ = -140.76 (d, 2F, J=16.1Hz, F(2,6)), -154.18 (t, 1F, J=20.7Hz, F(4)), -162.47 (t, J_1=19.8 Hz, J_2=16.7 Hz, F(3,5)).

^{13}C NMR ($CDCl_3$, 125.76 MHz): 165.33(s, C=O), 145.42(m, 1C, CF_{ar}), 141.19 (m, 1C, CF_{ar}), 139.19(m, 1C, CF_{ar}), 135.95(m, 1C, CF_{ar}), 108.37(m, 1C, CF_{ar}), 62.61 (s, CH) , 46.89 (s, CH_2), 13.63 (s, CH_3).

IR (KBr): 2989, 1747, 1515, 1312, 1228, 635 cm^{-1}.

HRMS (ESI+): m/z $[M]^+$ calcd for $C_{13}H_{11}F_5O_4$: 326.0577; found: 326.0584.

For $C_{13}H_{11}F_5O_4$ calcd, % : C, 47.86; H, 3.40; F, 29.12. Found, %: C, 47.92; H, 3.46; F, 29.47.

2-(Perfluorophenyl)acetic acid (12)

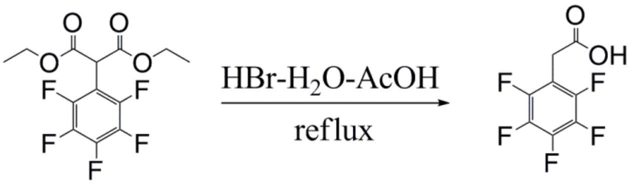

3 **12**

Diethyl 2-(perfluorophenyl)malonate (7.0 g, 21.5 mmol) was dissolved in a mixture of 48% aqueous HBr (15 mL) and glacial AcOH (75 mL). The resulting mixture was refluxed with magnetic stirring for 10 h until TLC (hexane:CH_2Cl_2, 1:4 v/v, silica gel) indicated complete consumption of the ester, and then cooled to room temperature. The solvents were evaporated under reduced pressure and the resulting solid was triturated with hexane (2 × 20 mL) and filtered. The light gray solid was dried in air and recrystallized from a heptane/CH_2Cl_2 mixture. The yield of 2-(perfluorophenyl)acetic acid as a white solid was 3.1 g (64%). M.p. 108–109 °C (lit. 109 °C [2])

^{1}H NMR ($CDCl_3$, 300 MHz): δ = 10.89 (broad s, 1H, OH), 3.81 (s, 2H, CH_2).

^{19}F NMR ($CDCl_3$, 282.5 MHz): δ = -143.05 (d, 2F, J=14.5 Hz, F(2,6)), -155.30 (t, 1F, J_1=J_2=20.5Hz, F(4)), -162.85 (td, 2F, J_{1d}=6.8 Hz, J_{2d}=13.1Hz, J_{3d}=7.1 Hz, J_{1t} = J_{2t}=13.8 Hz, F(3,5)).

^{13}C NMR ($CDCl_3$, 125.8 MHz): δ = 174.92 (s, 1C, C=O), 146.34 (m, 1C, CF_{ar}), 144.36 (m, 1C, CF_{ar}), 141.91(m, 1C, CF_{ar}), 139.81(m, 1C, CF_{ar}),138.57(m, 1C, CF_{ar}),136.50 (td, 1C, J_1=4.1Hz, J_2=18.3Hz, CH_2).

IR (KBr): 2978, 1717, 1510, 1248, 1025, 648 cm^{-1}.

HRMS (ESI+): m/z $[M+H]^+$ calcd for $C_8H_4F_5O_4$: 227.0131; found: 227.0138.

For $C_8H_3F_5O_4$ calcd, % : C, 42.50; H, 1.34; F, 42.01. Found, %: C, 47.77; H, 1.30; F, 42.23.

General method for the hydrolysis of ester (3)

A weighted portion of diethyl 2-(perfluorophenyl)malonate (usually 0.652 g, 2 mmol) was placed into a 50 mL screw cup vial (for experiments at rt) or into a 50 mL conical flack equipped with a reflux condenser. A measured volume of a liquid reagent (e.g., 10 mL of 15% aqueous solution of NaOH and 10 mL of Et_2O, etc.) was added and the mixture was vigorously stirred magnetically at a definite temperature. As far as TLC analysis was sometimes inefficient, other analytical methods were applied depending on the nature of the reagents.

For homogeneous alkaline water–organic solvent mixtures, the volatiles were removed in a rotary evaporator at room temperature. The remaining liquid was extracted several times with MTBE (methyl *tert*-butyl ether), the aqueous phase was acidified to pH =1 with HCl and evaporated to dryness. The dry residue was extracted with hot iPrOH and filtered, then the organic solvent was evaporated to dryness. The MTBE solution was dried over $MgSO_4$ and also evaporated to dryness at room temperature under reduced pressure. The acidic and neutral components separated in this way were weighted and analyzed by NMR.

The heterogeneous alkaline samples were first partitioned between water and MTBE, and the water and organic phases were treated as indicated above. The homogeneous organic alkaline samples were first evaporated and then treated like heterogeneous ones. The acidic samples were evaporated to dryness and partitioned between 5% aqueous NaOH solution and MTBE, then treated as described above.

Molecular structure of compound 12.

Experimental

Single crystals of **12** suitable for X-ray analysis were obtained by slow evaporation of a saturated solution of **12** in a hexane/dichloromethane mixture. The X-ray diffraction study of single crystals of **12** was performed on a Bruker Apex II diffractometer (CCD detector, Mo K_{α}, λ = 0.71073 Å, graphite monochromator) [3]. The structure was solved by direct methods and refined in the full-matrix anisotropic approximation for all non-hydrogen atoms. Hydrogen atoms at carbon atoms were generated geometrically and refined in the "riding" model. The H atoms at oxygen atoms were located in the difference Fourier maps. The calculations were performed using the SHELX-2018 software package [4]. The main crystallographic parameters and refinement details are as follows: $2(C_8H_3F_5O_2)$, *fw* = 452.21, colorless, parallelogram, crystal size 0.25 x 0.20 x 0.20, triclinic system, space group *P*-1, *a* = 4.8287(2)Å, *b* = 12.7925(7) Å, *c* = 14.3741(7) Å, α = 113.220(2)^o, β = 90.298(2)^o, γ = 92.372(2)^o, *V* = 815.06(7) Å^3, *Z* = 2, ρ_{calc} =

1.843 g·cm^{-3}, μ = 0.204 cm^{-1}, 9996 measured reflections, 3428 reflections with $I > 2.0\sigma(I)$, R_{int} = 0.0223, *GooF* = 0.981, R_1 ($I > 2\sigma(I)$) = 0.0564, wR_2 ($I > 2\sigma(I)$) = 0.1472, R_1 (all data) = 0.0833, wR_2 (all data) = 0.1666, $T_{min/max}$ = 0.6736/ 0.7461. The structure parameters were deposited with the Cambridge Structural Database (CCDC No. 1993963; http://www.ccdc.cam.ac.uk/).

Discussion

The molecules of **12** crystallize as two independent molecules with similar bond lengths and interatomic angles (average values C=O 1.220 Å, C-OH 1.303 Å, C(CH_2)-C 1.502 Å, C-C(arom) 1.374 Å, C-F 1.339 Å, O-C-O 124.85°, C(C_6)-C(CH_2)-C(CO_2) 114.20°). Each two molecules of the acid form a cyclic dimer as a result of double hydrogen bonding (O-H 0.88 Å, H…O 1.79 Å, O…O 2.657 Å, O-H-O 173.6°, Fig. S1) that is often observed for carboxylic acids in crystals [5].

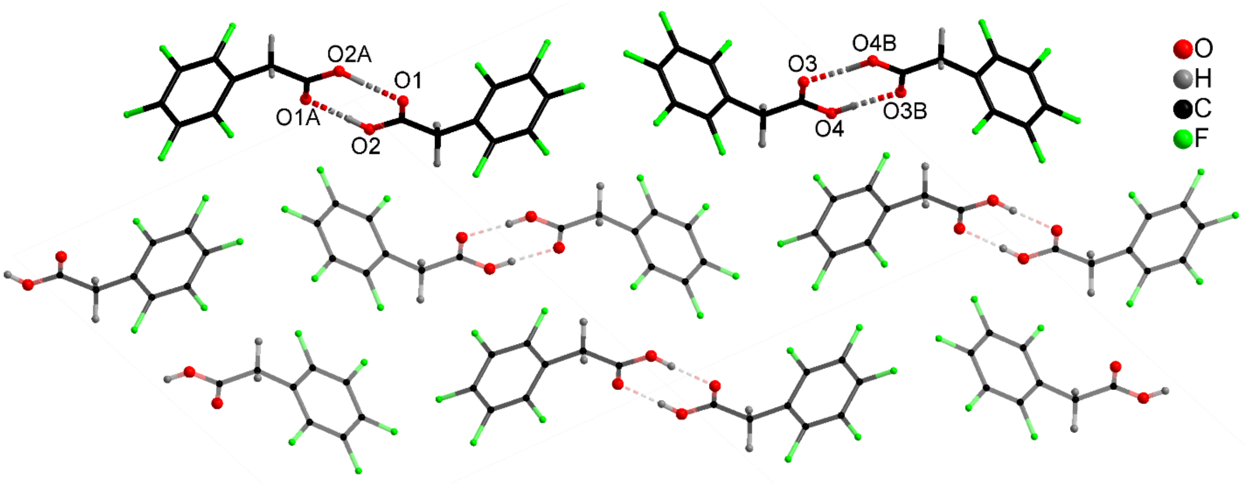

Figure S1. Crystal packing of **12** (atoms with additional letters in the atom labels are at (2-*x*, -*y*, 2-*z* (A)*;* 1-*x*, 2-*y*, 1-*z* (B).

References

1. Vlasov VM, Krivousova ED, Yakobson GG. *Zh Org Khim*. **1970**, 6,758–67. (CA 73:13946).

2. Barbour AK, Buxton MW, Coe PL, Stephens R, Tatlow JC. *J Chem Soc*. **1961**, 808-817.

3. *SMART (Control) and SAINT (Integration) Software*, Version 5.0, Bruker AXS Inc., Madison, WI, **1997**.

4. Sheldrik GM. *Acta Crystallogr.*, **2008**, A64, 112-122.

5. Gavezzotti A. *Acta Crystallogr.* B, **2008**, 64, 401-403

^{1}H NMR spectrum of **12**

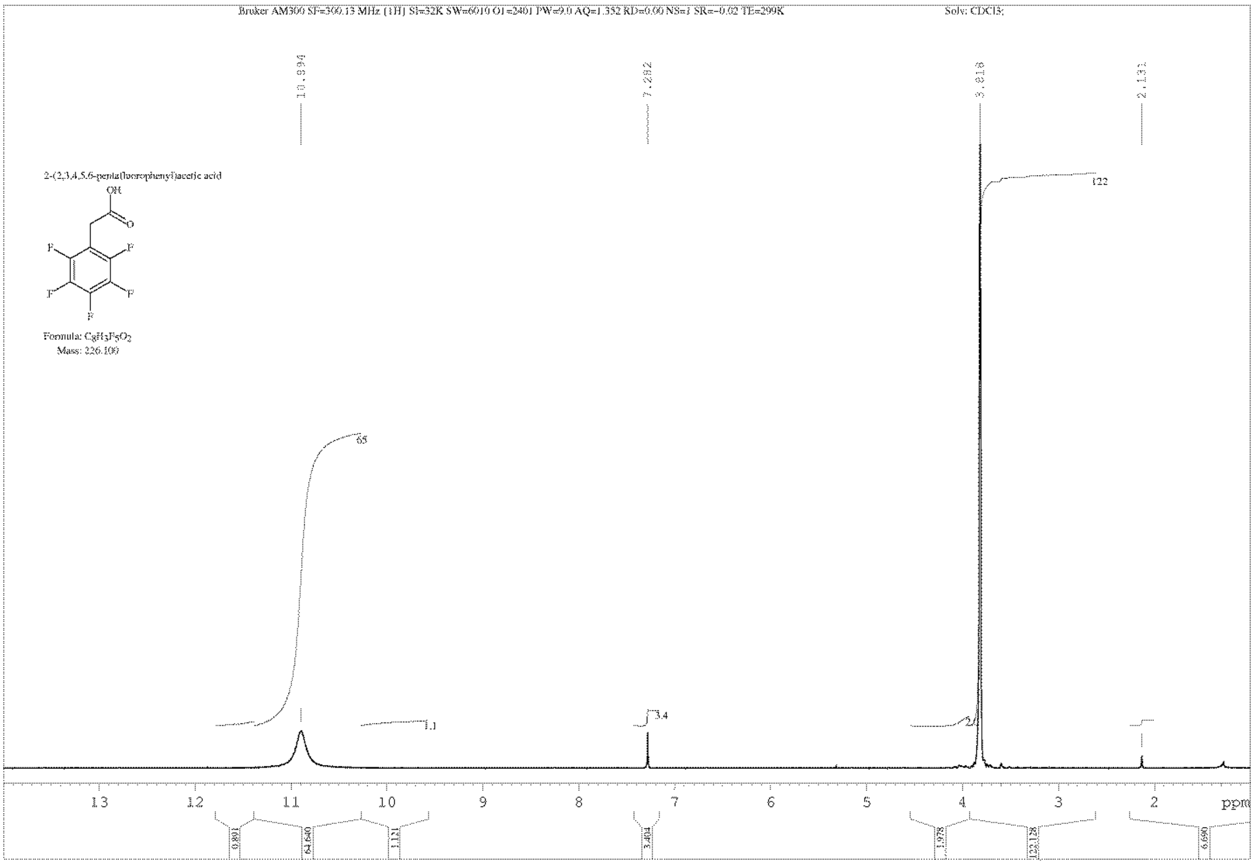

^{13}C NMR spectrum of **12**

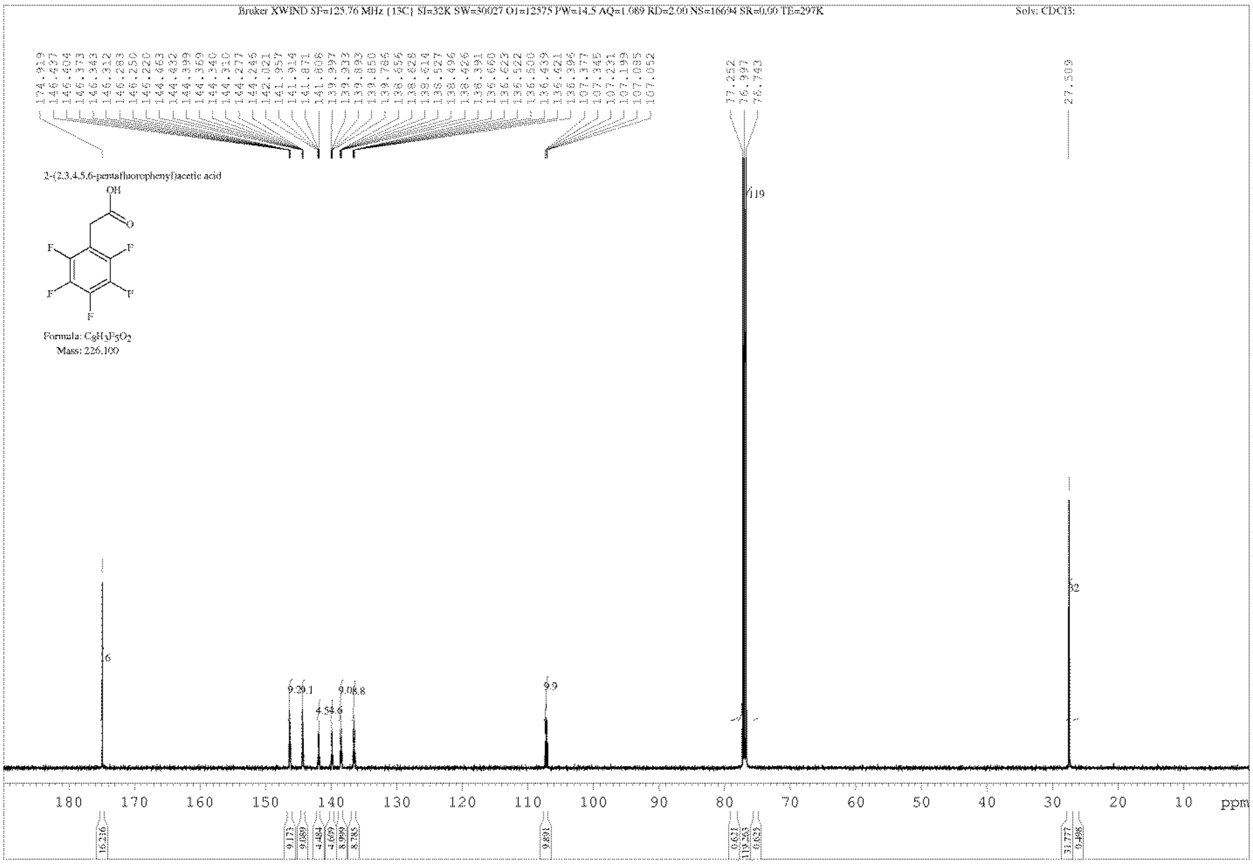

^{19}F NMR spectrum of **12**

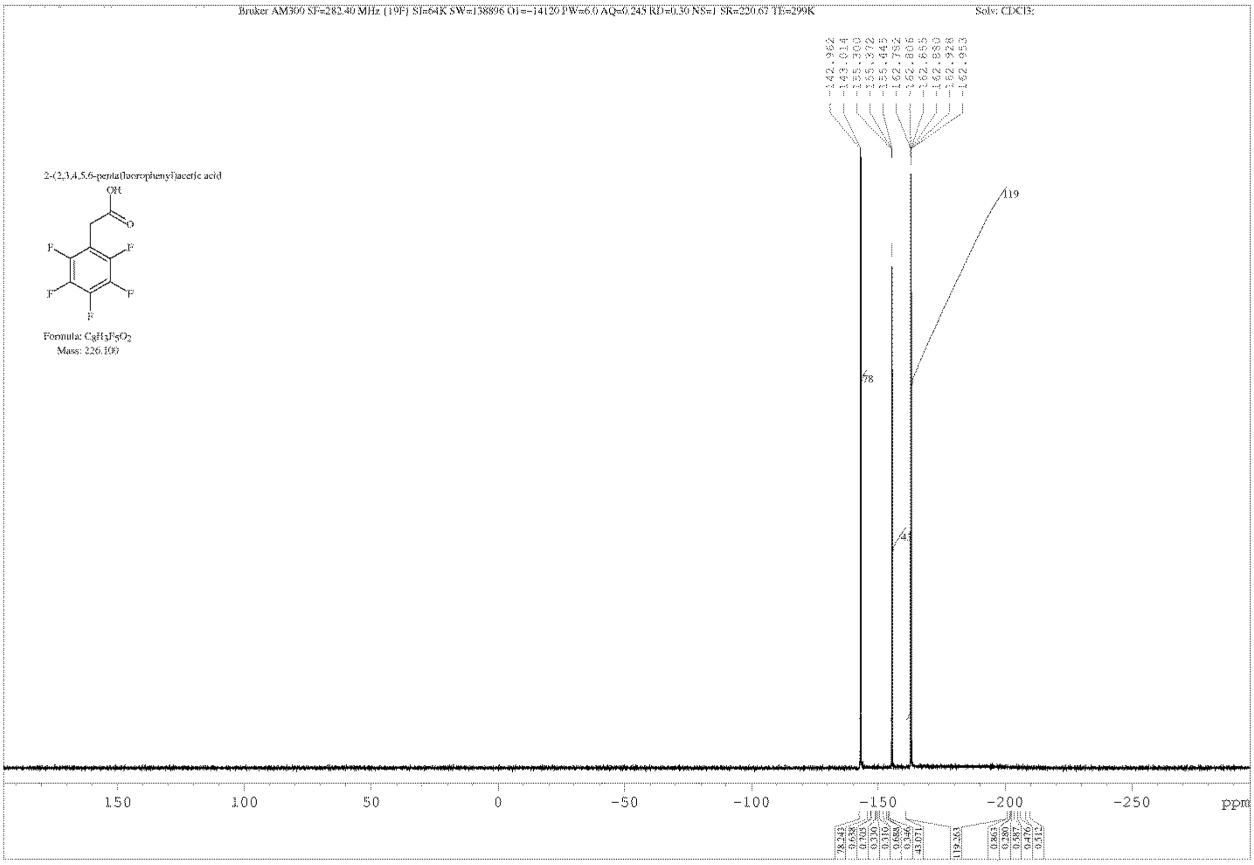

^{1}H NMR spectrum of **2**

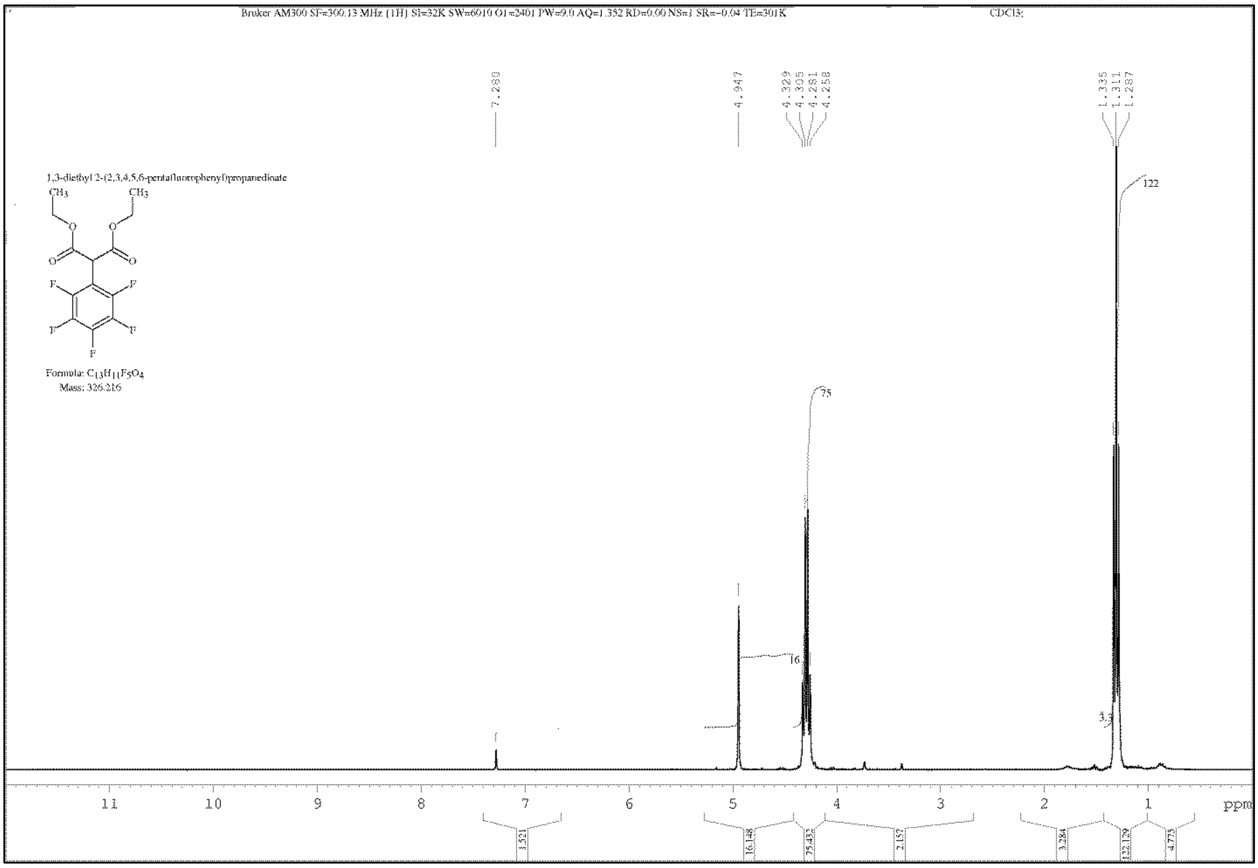

^{19}F NMR spectrum of **2**

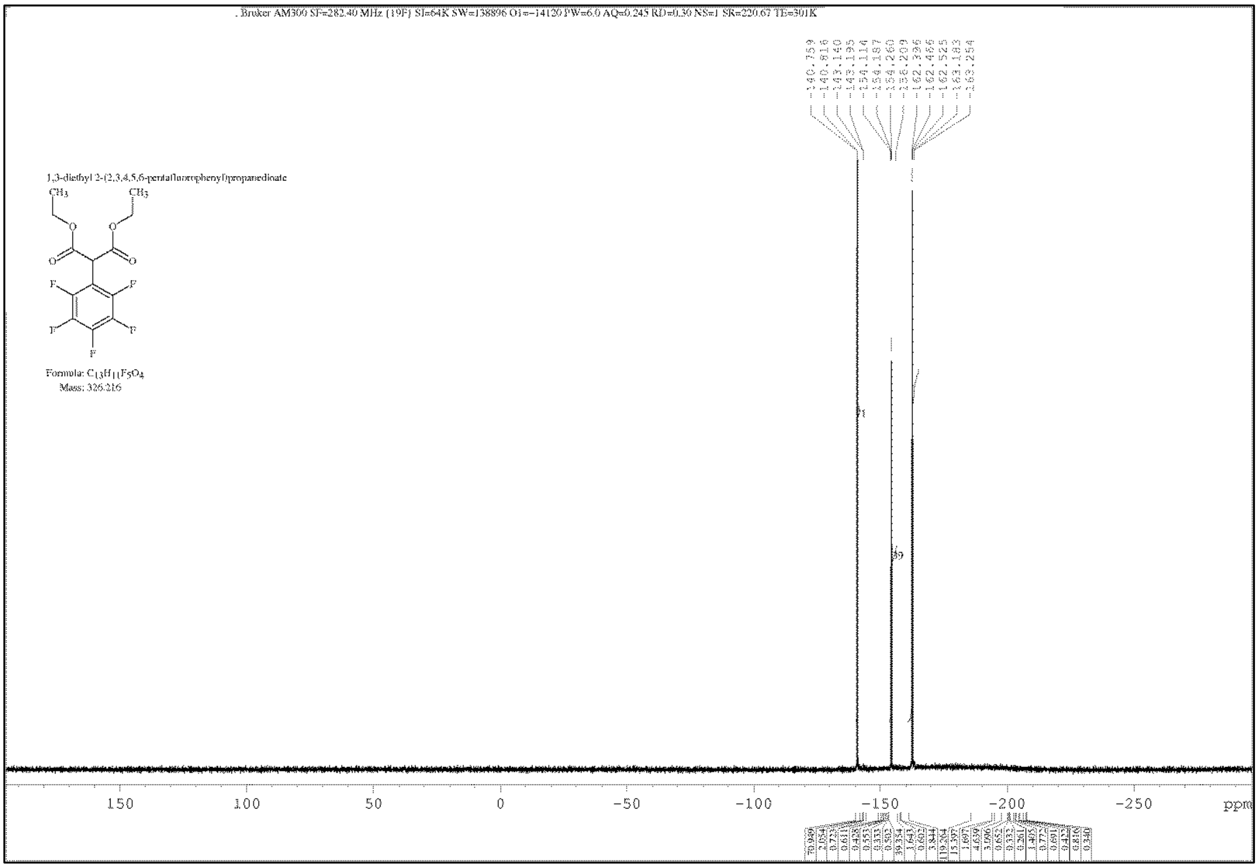

Supplement: File 1 — Detailed information about experimental procedures, X-ray diffraction experiments for compound 12 and characterization data for compounds 3 and 12. [file Beilstein_J_Org_Chem-16-1863-s001.pdf]
